# The Host E3-Ubiquitin Ligase TRIM28 Impedes Viral Protein GP4 Ubiquitination and Promotes PRRSV Replication

**DOI:** 10.3390/ijms241310965

**Published:** 2023-06-30

**Authors:** Zhiying Cui, Likun Zhou, Shijie Zhao, Wen Li, Jiahui Li, Jing Chen, Yina Zhang, Pingan Xia

**Affiliations:** 1 College of Life Science, Henan Agricultural University, Zhengdong New District Longzi Lake 15#, Zhengzhou 450046, China; 2 College of Veterinary Medicine, Henan Agricultural University, Zhengdong New District Longzi Lake 15#, Zhengzhou 450046, China

**Keywords:** TRIM28, PRRSV, GP4, ubiquitination, virus-host interactions

## Abstract

Porcine reproductive and respiratory syndrome (PRRS), caused by the PRRS virus (PRRSV), is a highly pathogenic porcine virus that brings tremendous economic losses to the global swine industry. PRRSVs have evolved multiple elegant strategies to manipulate the host proteins and circumvent against the antiviral responses to establish infection. Therefore, the identification of virus–host interactions is critical for understanding the pathogenesis of PRRSVs. Tripartite motif protein 28 (TRIM28) is a transcriptional co-repressor involved in the regulation of viral and cellular transcriptional programs; however, its precise role in regulating PRRSV infection remains unknown. In this study, we found that the mRNA and protein levels of TRIM28 were up-regulated in PRRSV-infected porcine alveolar macrophages (PAMs) and MARC-145 cells. Ectopic TRIM28 expression dramatically increased viral yields, whereas the siRNA-mediated knockdown of TRIM28 significantly inhibited PRRSV replication. Furthermore, we used a co-immunoprecipitation (co-IP) assay to demonstrate that TRIM28 interacted with envelope glycoprotein 4 (GP4) among PRRSV viral proteins. Intriguingly, TRIM28 inhibited the degradation of PRRSV GP4 by impeding its ubiquitination. Taken together, our work provides evidence that the host E3-ubiquitin ligase TRIM28 suppresses GP4 ubiquitination and is important for efficient virus replication. Therefore, our study identifies a new host factor, TRIM28, as a potential target in the development of anti-viral drugs against PRRSV.

## 1. Introduction

The porcine reproductive and respiratory syndrome virus (PRRSV), which is a highly contagious pathogen that causes reproductive disorders and severe dyspnea in pigs, has been regarded as a persistent challenge for the swine industry globally [1]. The PRRSV has been recently classified as PRRSV-1 (species Betaarterivirus suid 1) and PRRSV-2 (species Betaarterivirus suid 2) [2]. The genome of the PRRSV is approximately 15.4 kb with more than eleven open reading frames (ORFs), encoding eight structural proteins (GP2a, GP2b, GP3, GP4, GP5, GP5a, M, and N) and at least sixteen non-structural proteins (NSP1α, NSP1β, NSP2-6, NSP-2N, NSP-2TF, NSP7α, NSP7β, and NSP8-12) [3,4]. The structural envelope glycoprotein 4 (GP4) plays a crucial role in generating infectious PRRSVs [5]. Previous research has identified that GP4 contributes to inducing protective immune responses [6,7,8]. More importantly, GP4 co-localizes with cluster of differentiation 163 (CD163), which is a major receptor of PRRSV attachment, thus mediating the virus entry process [9]. Specifically, the GP2a, GP3, GP4, and GP5 proteins form a heterotetrameric complex that is required to transport these proteins from the endoplasmic reticulum (ER) to the Golgi apparatus in each infected cell prior to virion assembly [10]. It has not been explored that the viral structural proteins utilize the interaction between host factors and viral proteins to facilitate PRRSV replication.

The ubiquitination of proteins is a posttranslational modification (PTMs) process with many cellular functions including the regulation of virus replication [11]. Tripartite motif (TRIM) proteins are a large family of E3 ubiquitin ligases that are implicated in multiple biological processes ranging from transcriptional regulation to posttranslational modification [12,13]. TRIM proteins have been shown to mediate the transfer of ubiquitin to target proteins, especially a number of viral proteins identified as the substrates of TRIM proteins during virus infection [14]. Many TRIM proteins are known to inhibit viral replication [15,16]; however, very few examples exist of TRIM proteins being exploited by viruses to promote virus replication [17,18].

TRIM28 (also known as Kruppel-associated box-associated protein 1 (KAP1), or transcription intermediary factor 1β (TIF1β)) belongs to a subset of TRIM proteins called the transcription intermediary factor 1 (TIF1) sub-family. TRIM28 is characterized by a conserved N-terminal architecture consisting of a Really Interesting New Gene (RING) E3 ubiquitin ligase domain (R), two B-box domains (B) involved in higher-order oligomerization, and one coiled-coil (CC) domain required for dimerization, collectively known as the RBCC domain. Its C-terminus contains a plant homeodomain (PHD) involved in an intramolecular small ubiquitin-related modifier (SUMO) E3 ligase and a bromodomain (BR), and the SUMOylation of the PHD-BR is required for TRIM28’s repressive activity [19]. This RBCC-PHD-BD structure is a characteristic only shared by the three other TRIM-family members: TRIM24/TIF1α, TRIM33/TIF1γ, and TRIM66/TIF1δ. All four proteins have been known for their function as transcriptional regulators [20,21]. Recent reports have demonstrated that TRIM28 could regulate protein posttranslational modification and is involved in the process of viral infection [22]. However, its effects on the posttranslational modification of viral proteins have not been elucidated. In this report, we identified that a host interactor, TRIM28, directly targets PRRSV viral protein GP4 and inhibits its ubiquitination, which protects GP4 protein from degradation and promotes PRRSV replication. Therefore, our data suggest that the TRIM28-mediated inhibition of viral protein ubiquitination may represent an escape mechanism by which the virus utilizes host factors to facilitate viral protein stabilization and expression.

## 2. Results

### 2.1. TRIM28 Is Induced by PRRSV Infection

**Figure 1:**
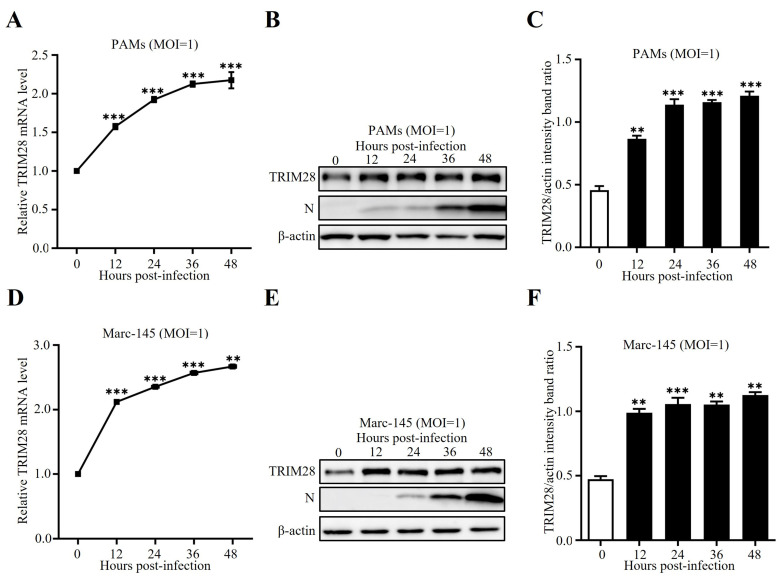
PRRSV infection induces the expression of TRIM28. PAMs (**A**–**C**) or MARC-145 cells (**D**–**F**) were infected with PRRSVs at a multiplicity of infection (MOI) of 1 for 0, 12, 24, 36, or 48 hpi, respectively. The cells were collected, and then the mRNA (**A**,**D**) and protein (**B**,**E**) levels of TRIM28 were detected by using RT-qPCRs and Western blotting analysis. Error bars: means ± SDs of 3 independent tests. Student’s *t*-test: ** *p* < 0.01; *** *p* < 0.001 compared to control.

To evaluate how TRIM28 responds to PRRSV infection, we first measured TRIM28 expression in porcine alveolar macrophages (PAMs) infected with PRRSVs at an MOI of 1 for 0, 12, 24, 36, or 48 h. The mRNA and protein levels of TRIM28 were dramatically induced during PRRSV infection compared with those in uninfected cells (Figure 1A–C). In agreement with our observations in PAMs, we found that PRRSV infection greatly increased the mRNA and protein levels of TRIM28 in MARC-145 cells (Figure 1D–F). Taken together, these data suggest that PRRSV infection could up-regulate TRIM28 expression. 

### 2.2. TRIM28 Overexpression Facilitates PRRSV Replication

**Figure 2:**
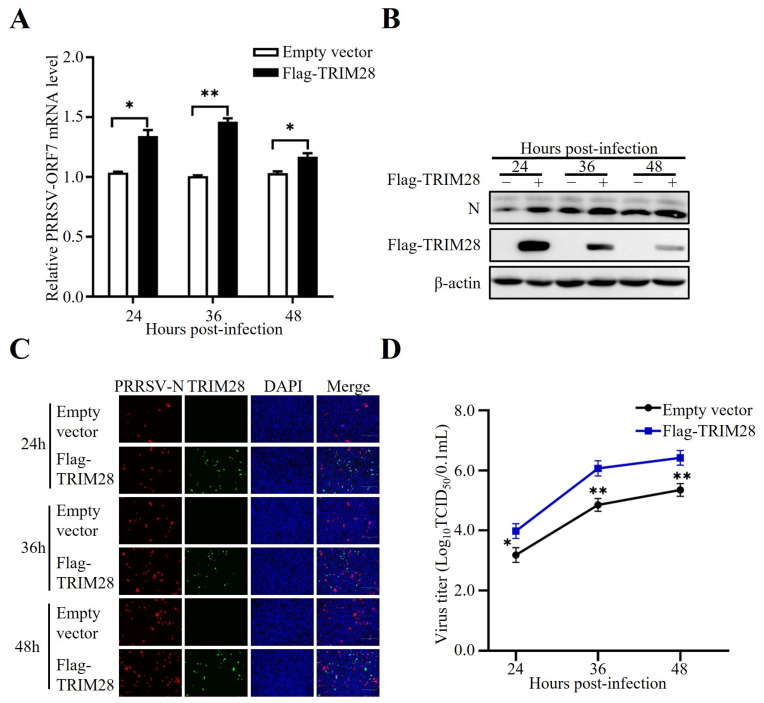
TRIM28 overexpression facilitates PRRSV replication. After being transfected for 24 h with Flag-TRIM28 or empty vector (EV), MARC-145 cells were infected with PRRSV for 24, 36, or 48 h. Virus RNA levels (**A**, RT-qPCR), PRRSV-N protein expression (**B**, Western blotting; **C**, IFA—the cells were imaged for PRRSV-N (red) and TRIM28 (green). Their nuclei were stained with DAPI. Scale bar: 300 μm), and virus titers (**D**, TCID50) were detected. Error bars: means ± SDs of 3 independent tests. Student’s *t*-test: * *p* < 0.05; ** *p* < 0.01 compared to control.

To explore whether TRIM28 could affect PRRSV replication, PRRSV infection assays were performed in MARC-145 cells transfected with overexpressions of TRIM28 expression construct. PRRSV infection was examined by using Western blotting and immunofluorescence assay (IFA) analysis using Abs against PRRSV nucleocapsid protein N. PRRSV RNA levels were analyzed using RT-qPCRs with specific primers detecting ORF7. The amounts of PRRSV production were measured by using TCID_50_. The results showed that ectopically expressed TRIM28 dramatically increased not only PRRSV infection but also the abundance of viral RNAs (Figure 2A), viral N protein (Figure 2B,C), and virus titers (Figure 2D). Together, these data indicate that overexpressed TRIM28 could promote PRRSV replication.

### 2.3. TRIM28 Knockdown Inhibits PRRSV Replication

**Figure 3:**
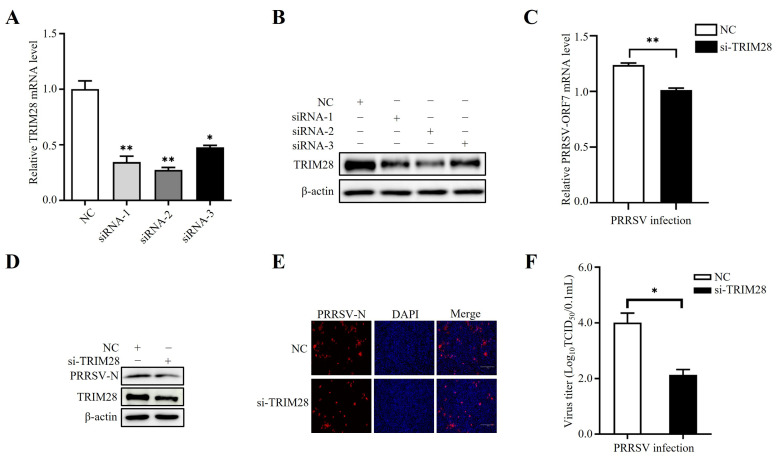
TRIM28 knockdown inhibits PRRSV replication. (**A**,**B**) The knockdown efficiency of siRNA-TRIM28. Three siRNAs that target TRIM28 or negative control siRNA (NC) were transfected into MARC-145 cells for 48 h. RT-qPCRs and Western blotting analyses were used to identify the expression of TRIM28 mRNA and protein expression, respectively. (**C**–**F**) Effect of TRIM28 knockdown on PRRSV replication. Si-TRIM28 or NC were transfected into MARC-145 cells for 48 h, and the cells were infected with PRRSVs. Virus RNA levels (**C**), expression of PRRSV-N protein (**D**, Western blotting; **E**, IFA—the cells were imaged for PRRSV-N (red). Their nuclei were stained with DAPI. Scale bar: 300 μm. The scale bars in the images were added by the Image J software, version 1.8), and virus titers (**F**) were detected. Error bars: means ± SDs of 3 independent tests. Student’s *t*-test: * *p* < 0.05; ** *p* < 0.01 compared to control.

To further investigate the function of TRIM28 in PRRSV infection, small interfering RNA (siRNA)-knockdown experiments were performed. Synthesized siRNA targeting TRIM28 was used to suppress endogenous TRIM28 expression in MARC-145 cells (Figure 3A,B). The SiRNA-mediated knockdown of TRIM28 expression significantly reduced not only PRRSV infection but also the abundance of viral RNAs (Figure 3C), viral N protein (Figure 3D,E), and virus titers (Figure 3F). Collectively, these results demonstrate that TRIM28 knockdown could suppress PRRSV infection.

### 2.4. TRIM28 Targets PRRSV GP4

**Figure 4:**
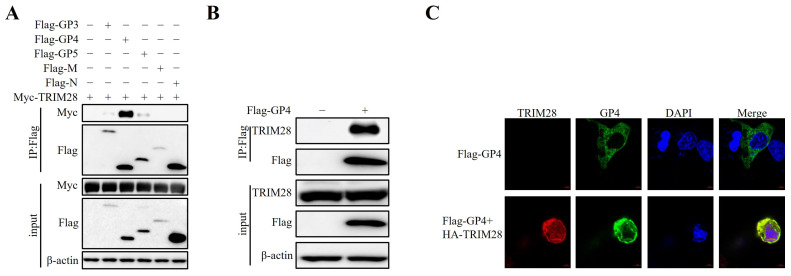
TRIM28 interacts with PRRSV GP4. (**A**) Analysis of TRIM28-interacting PRRSV viral proteins. HEK293T cells were transfected with Myc-TRIM28 along with Flag-tagged PRRSV viral proteins (GP3, GP4, GP5, M, and N) and a co-IP assay was performed by anti-Flag mAb. (**B**) Co-IP analysis of the interaction between TRIM28 and GP4. MARC-145 cells were transfected with Flag-GP4. Cell lysates were prepared at 36 h post-transfection, and endogenous TRIM28 was immunoprecipitated using the anti-Flag antibody, followed by immunoblotting with anti-TRIM28. (**C**) Co-localization analysis of TRIM28 and GP4. MARC-145 cells were co-transfected with HA-TRIM28 and Flag-GP4 for 48 h. Cells were fixed and observed under a confocal microscopy; the cells were imaged for TRIM28 (red) and GP4 (green). Their nuclei were stained with DAPI. Scale bar: 5 μm. The scale bars in the images were added by ZEN 3.4 (blue edition).

Our results above confirmed that TRIM28 plays an important role in PRRSV replication. Recent studies have shown that TRIM proteins are involved in the regulation of viral infection by targeting viral proteins [16,17,18]. We therefore investigated whether TRIM28 interacts with PRRSV viral proteins. Recombinant expression vectors of TRIM28 and PRRSV protein were transfected into HEK293T cells, and co-immunoprecipitation (co-IP) analysis indicated that TRIM28 was associated clearly with GP4 and weakly with GP3 and GP5, but not with M or N (Figure 4A). To confirm the interaction of TRIM28 with PRRSV GP4, the Flag-GP4 expression plasmid was transfected into MARC-145 cells. An anti-Flag antibody was used for co-IP analysis, and an anti-TRIM28 antibody was used to detect endogenous TRIM28 by means of Western blotting. As shown in Figure 4B, ectopically expressed GP4 co-precipitated endogenous TRIM28. Subsequently, we performed a confocal microscopy analysis to investigate whether TRIM28 and GP4 co-localize at similar subcellular positions. The results showed that TRIM28 was localized in both the nucleus and cytoplasm, whereas PRRSV GP4 was mostly distributed in the cytoplasm and TRIM28 and GP4 were remarkably co-localized in the perikaryon (Figure 4C). Taken together, these data suggest that TRIM28 could interact with PRRSV GP4.

### 2.5. TRIM28 Inhibits the Degradation of PRRSV GP4 by Impeding Its Ubiquitination

**Figure 5:**
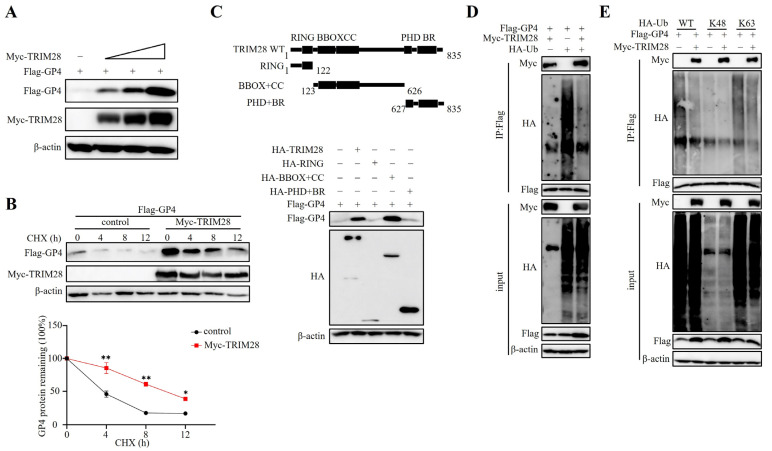
TRIM28 inhibits the degradation of PRRSV GP4 by impeding its ubiquitination. (**A**) Effect of TRIM28 on GP4 expression. HEK293T cells were transfected with Flag-GP4 and increasing amount of Myc-TRIM28 plasmid. The cellular extracts were then subjected to Western blotting using the indicated antibodies. (**B**) Effect of TRIM28 on GP4 half-life. HEK293T cells were co-transfected with Myc-TRIM28 and Flag-GP4, then treated with CHX (100 μg/mL) for the indicated times after 24 h transfection. The cell samples were subjected to immunoblotting analysis with the indicated antibodies. (**C**) Schematic depiction of TRIM28 motif and its deletion mutants on the top. RING: a Really Interesting New Gene domain; BBOX: two B-box domains; CC: one antiparallel coiled-coil domain; PHD: a plant homeodomain; BR: a bromodomain. HEK293T cells were co-transfected with plasmids expressing domain-deleted TRIM28 mutants and GP4. Cell lysates were prepared at 36 h post-transfection and then subjected to Western blotting using the indicated antibodies. (**D**,**E**) Effect of TRIM28 on GP4 polyubiquitination modification. (**D**) HEK293T cells were co-transfected with Flag-GP4, HA-Ub (Ub: ubiquitin), and Myc-TRIM28 for 36 h. Cells were treated with MG132 (20 μM) for 6 h before collection. The cell lysates were subjected to analysis of Flag-precipitation and immunoblotting analysis. (**E**) HEK293T cells were co-transfected with Flag-GP4, Ub-HA (WT), Ub-HA (K48) or Ub-HA (K63), and Myc-TRIM28 for 36 h. Cells were treated with MG132 (20 μM) for 6 h before collection. The cell lysates were subjected to the analysis of Flag-precipitation and immunoblotting analysis. Error bars: means ± SDs of 3 independent tests. Student’s *t*-test: * *p* < 0.05; ** *p* < 0.01 compared to control.

Our results above indicate that TRIM28 interacts with PRRSV GP4. Since TRIM28 is a member of the TRIM family, which has E3 ubiquitin ligase activity, we speculated that TRIM28 may affect the expression level of PRRSV GP4. To test this hypothesis, the TRIM28 and GP4 expression plasmids were co-transfected into HEK293T cells. As shown in Figure 5A, the co-expression of gradually increasing amounts of TRIM28 increased the protein expression level of GP4 in a dose-dependent manner. Additionally, we performed a time course experiment to monitor Flag-GP4 degradation in the presence of cycloheximide (CHX) to inhibit protein synthesis. The overexpression of TRIM28 significantly slowed the degradation of GP4 (Figure 5B). These results demonstrate that TRIM28 stabilizes the GP4 protein. TRIM28 is composed of a RING finger domain, B-box domain, coiled-coil domain, PHD domain, and BR domain. To determine which domain of TRIM28 is essential for promoting GP4 expression, the vectors expressing domain-truncated TRIM28 mutants, TRIM28 (RING), TRIM28 (BBOX + CC), and TRIM28 (PHD + BR) were constructed and each TRIM28 mutant was co-transfected into the HEK293T cells together with Flag-GP4. As shown in Figure 5C, the truncation of the BBOX + CC domain of TRIM28 significantly up-regulated GP4 expression, indicating that the BBOX + CC domain of TRIM28 plays an essential role in increasing GP4 expression. We next sought to explore the mechanism of GP4 stabilization by TRIM28. TRIM28 functions as an E3 ligase. We performed a ubiquitination assay. HEK293T cells were transfected with HA-ubiquitin and Flag-GP4 in the absence or presence of TRIM28. As shown in Figure 5D, the poly-ubiquitination of PRRSV GP4 was significantly inhibited by TRIM28. To further determine which lysine residue ubiquitinations of PRRSV GP4 are suppressed by TRIM28, we used two ubiquitin mutants, K48 and K63, as substrates of ubiquitination. TRIM28 inhibited both the total and K63-linked ubiquitination of PRRSV GP4, whereas it had no effects on the K48-linked ubiquitination of PRRSV GP4 (Figure 5E). These results suggest that TRIM28 selectively attenuates the K63-linked ubiquitination of GP4 and its degradation.

## 3. Discussion

Presently, the majority of research papers on TRIMs have concentrated on how they operate as antiviral agents, either directly limiting viral replication or indirectly eliciting an antiviral innate immune response. However, it is unclear whether TRIMs can serve as “pro-viral” factors: that is, host components necessary for virus replication. According to several studies, some viral antagonists can exploit TRIMs to initiate their IFN antagonist action (TRIM23, for instance, ubiquitinates YFV-NS5 to inhibit STAT2 function [23]), but these are unintended consequences that provide viruses with an advantage by lowering host antiviral responses. It would not be surprising if TRIMs were involved in directly encouraging virus replication via non-degradative ubiquitinating viral proteins, given that the ubiquitination of viral proteins may positively affect particular steps of the replication cycle. Most viruses, including the PRRSV, interact with host proteins and utilize them to escape the antiviral response and fulfill virus replication and persistent infection. Our group discovered that TRIM28 was possibly involved in the regulation of PRRSV infection by using mass spectrometry. In the present study, our findings suggest that TRIM28 is a host factor targeted by PRRSV viral protein GP4 and that it directly enhances virus replication via inhibiting the K63-linked ubiquitination of GP4.

To verify whether TRIM28 is an effective target for PRRSV infection, we examined the possible correlation between TRIM28 expression and PRRSV infection progression. In this study, PRRSV infection induced TRIM28 gene expression in porcine alveolar macrophages (PAMs) and MARC-145 cells, indicating that PRRSVs might manipulate the host protein TRIM28 to facilitate their propagation. Nevertheless, the involved pathways, as well as the underlying mechanisms regulating TRIM28 expression during viral infection, have not yet been resolved. Previous studies have discovered that TRIM28 posttranslational modification (such as via phosphorylation and SUMOylation) is dramatically altered during virus infection, including the human adenovirus (HAdV) [24], influenza virus [25], human cytomegalovirus (HCMV) [26], kaposi’s sarcoma-associated herpesvirus (KSHV) [27], and merkel cell polyomavirus (MCPyV) [28]. It should be further explored whether PRRSV infection causes changes in the posttranscriptional modification of TRIM28.

Functionally, TRIM28 is frequently described as an important scaffold protein concentrated in gene promoter regions to restrict transcription [29,30]. This detrimental role of TRIM28 in gene transcription has significant consequences for viral transcription and replication. TRIM28 has previously been shown to repress viral transcription for several herpesviruses including the KSHV, HCMV, and Epstein–Barr virus (EBV) [26,31,32]. TRIM28 has also been shown to inhibit HIV-1 replication by binding the acetylated HIV-1 integrase and preventing the integration of pro-viral DNA [33] and to mediate the transcription suppression of the HIV-1 LTR promoter [34,35]. According to one recent study, TRIM28 inhibits Tas-dependent transactivation activity in prototype foamy virus (PFV) promoters, which limits PFV transcription and replication [36]. In this study, we found that ectopically expressed TRIM28 facilitated PRRSV replication while TRIM28 deficiency inhibited PRRSV replication, indicating that TRIM28 could promote PRRSV replication. These results indicated that TRIM28 may be a critical positive regulator for PRRSV infection. Therefore, we speculated that the role of TRIM28 in regulating PRRSV infection may be independent of its transcriptional inhibition. 

TRIM proteins have previously been reported to directly regulate viral proteins during viral infections. The PRRSV minor structural proteins GP2, GP3, and GP4 form non-covalent heterotrimers in the virion. The PRRSV GP4 protein is an important component in the formation of the viral replication complex, which is required for replication. As a result, research on the effects of PRRSV GP4 will reveal insights into the mechanistic replication of the PRRSV. Our results showed that TRIM28 interacted with the PRRSV viral protein GP4. Our further study confirmed that TRIM28 inhibited the K63-linked poly-ubiquitination of PRRSV GP4 and enhanced its protein expression. To our knowledge, this was the first time TRIM28 was identified as being involved in the stabilization of PRRSV viral protein expression. Multiple previous studies have shown that TRIM28 enhances the stability of substrate protein via SUMOylation [37,38,39,40]. A recent study has revealed that the E3 SUMO ligase TRIM28 facilitates the SUMO1 and SUMO2/3-catalyzed SUMOylation of NLRP3, whereby it attenuates the K48-linked ubiquitination of NLRP3, resulting in the enhancement of NLRP3 stability [41]. We did not test the SUMOylation of PRRSV GP4 by TRIM28, which was a shortcoming of this study. We speculated that SUMOylation mediated by TRIM28 may block the ubiquitination site of PRRSV GP4 and thus inhibit PRRSV GP4 degradation. Further analysis of the SUMOylation and ubiquitination sites of PRRSV GP4 will help elucidate the underlying mechanisms.

The PRRSV is a highly pathogenic porcine virus that causes significant economic losses in the global swine industry. In order to establish infection, the PRRSV has evolved numerous sophisticated methods to influence host proteins and evade antiviral responses. Our results showed that TRIM28 interacted with GP4 and reduced its K63-linked poly-ubiquitination, resulting in increased protein expression, which aided virus propagation. As a result, we can develop inhibitors of TRIM28 to reduce the viral load of PRRSVs that can be used in the treatment of PRRSVs.

## 4. Materials and Methods

### 4.1. Cells and Virus Strain 

PAMs were collected via the bronchoalveolar lavage method from healthy six-week-old Large White–Dutch Landrace crossbred piglets as previously described [42] and maintained in RPMI-1640 medium supplemented with 10% FBS at 37 °C with 5% CO_2_. MARC-145 and HEK-293T cells were cultured in Dulbecco’s modified Eagle’s medium containing 10% FBS at 37 °C with 5% CO_2_. PRRSV strain HN07-1 (GenBank accession number KX766378.1) was propagated in the MARC-145 cells. PRRSV titers were measured by means of a microtitration assay using MARC-145 cells in 96-well plates and calculated as 50% tissue culture infective doses (TCID_50_) per milliliter according to the method of Reed and Muench.

### 4.2. Virus Infection 

The PAMs and MARC-145 cells were grown to approximately 70% to 80% confluence and infected with PRRSV strain HN07-1 at an MOI of 1 for 2 h. Then, the supernatants were removed and the cell monolayers were rinsed with PBS to remove un-attached virus particles. After that, the cells were incubated in fresh medium containing 2% FBS at 37 °C and 5% CO_2_ for 0, 12, 24, 36, or 48 h.

### 4.3. Antibodies and Reagents 

Antibodies for TRIM28 (15202-1-AP) and HRP-β-actin (HRP-60008) were procured from Proteintech. Antibodies for Flag (14793) and Myc (2272) were procured from CST. The antibody for HA (H3663) was procured from Sigma. FITC-labeled goat anti-rabbit IgG (H + L) (5230-0426), FITC-labeled goat anti-mouse IgG(H + L) (5230-0427), KPL Peroxidase-Labeled Antibody To Mouse IgG(H + L) (5220-0341), and KPL Peroxidase-Labeled Antibody To Rabbit IgG(H + L) (5220-0336) were procured from seracare. Alexa Fluor^TM^ 546 donkey anti-rabbit IgG (H + L) (A10040) and Alexa Fluor^TM^ 546 donkey anti-mouse IgG (H + L) (A11030) were procured from Invitrogen (Waltham, MA, USA). Chloroquine (C6628), DMSO (D8418), and poly (I:C) (P0913) were purchased from Sigma-Aldrich (St. Louis, MO, USA). Chloroquine and poly (I:C) were used at final concentrations of 100 μM and 10 μg/mL, respectively. The ANTI-FLAG^®^ M2 Affinity Gel (A2220) used for IP was purchased from Sigma-Aldrich. MG132 (S1748), NP-40 Lysis Buffer (P0013F), PMSF Solution (100 mM) (ST507), and Protein A + G Agarose (P2055), used for IP, were obtained from Beyotime (Shanghai, China). MG132 was used at a final concentration of 20 μM. RNA isolater Total RNA Extraction Reagent (R401-01), HiScript II Q RT SuperMix for qPCR (+gDNA wiper) (R223-01), and ChamQ Universal SYBR qPCR Master Mix(Q711-02) were procured from Vazyme (Nanjing, China). The Dulbecco’s modified Eagle’s medium and RPMI-1640 medium were procured from Hyclone (Logan, UT, USA). Fetal Bovine Serum (FBS) was procured from Gibco (Grand Island, NY, USA).

### 4.4. Expression Vector Construction and Plasmid Transfection

The full-length sequences of TRIM28 (GenBank accession numbers XM_007998459.2) cDNA were obtained by using an RT-PCR and cloned into the mammalian expression vectors pCMV-Flag-N, pCMV-HA-N, or pCMV-Myc-N. Various truncated plasmids of TRIM28 were generated from corresponding wild-type constructs. The recombinant plasmids of the Flag-tagged PRRSV viral proteins (GP3, GP4, GP5, M, and N) were preserved in our laboratory. All the specific primers used for plasmid construction were designed using Primer Premier 5 and listed in Table 1. All constructs were confirmed by DNA sequencing. Lipofectamine 2000 was used to transfect MARC-145 or HEK293T cells with recombinant expression vectors as directed by the manufacturer.

### 4.5. RNA Interference

Small interfering RNAs (siRNAs) that targeted TRIM28 (GenBank no. XM_007998459.2) were synthesized by GenePharma. MARC-145 cells were seeded in 6-well plates and transfected with 50 pM siTRIM28 or negative-control siRNA (NC) using Lipofectamine 2000 according to the manufacturer’s instructions for 48 h. The effects of siRNAs were analyzed by using RT-qPCRs and Western blotting. Table 2 shows the sequences of the siRNAs.

### 4.6. RNA Isolation and RT-qPCRs

Total RNA was extracted by an RNA extraction kit according to the instructions, and the purity and concentration of RNA were detected by a spectrophotometer. In each reaction system, 1 ug RNA was reverse-transcribed into 20 uL cDNA according to the protocol for a subsequent RT-qPCR assay. RT-qPCRs were performed using SYBR green PCR mix on a CFX96^TM^ Real-Time System. A single cycle of denaturation at 95 °C for 30 s was followed by 40 cycles of amplification at 95 °C for 5 s and 60 °C for 34 s. In order to confirm product specificity, a final melting cycle was added to create a melting curve. A single peak obtained in the melting curve verified the specificities of the PCR products. The housekeeping gene β-actin was used to standardize the relative gene expression levels. The number of fold changes in the levels of gene expression was calculated using the 2^−ΔΔct^ method. Table 1 lists all of the primers for RT-qPCRs, among which primers Swine-β-actin-For, Swine-β-actin-Rev, PRRSV-ORF7-For and PRRSV-ORF7-Rev are from the references and the other primers were designed by Primer Premier 5.

### 4.7. Western Blotting

Cell samples were lysed using lysis buffer (5% SDS and 1%TritonX-100) to extract total proteins and boiled in 4× loading buffer for 10 min at 100 °C. The cell lysates were subjected to SDS–PAGE. The proteins were transferred to nitrocellulose filter (NC) membranes and reacted with the appropriate antibodies (anti-TRIM28, dilution 1:2000; anti-PRRSV-N, dilution 1:200; anti-β-actin, anti-Flag, anti-Myc, and anti-HA were diluted with 1:1000; secondary goat anti-mouse, dilution 1:5000; secondary goat anti-rabbit, dilution 1:8000). Signals were detected with an ultrasensitive ECL Chemiluminescence Detection Kit.

### 4.8. Immunofluorescence Assay (IFA)

Cells in culture plates were first fixed with 4% paraformaldehyde for 30 min and then permeabilized with 0.1% TritonX-100 in PBS for 10 min. The fixed cells were washed with PBS and blocked with 5% BSA in PBS containing 0.1% Tween-20 for 1 h to prevent nonspecific binding. The cells were stained with specific primary antibodies (anti-Flag was diluted with 1:500; anti-HA and anti-PRRSV-N were diluted with 1:100), followed by blotting with fluorescent conjugated secondary antibody (FITC-labeled secondary antibodies were diluted with 1:400; Alexa Fluor^TM^ 546 labeled donkey anti-rabbit and donkey anti-mouse IgG were diluted with 1:500) in the dark for 1 h. The cellular nuclei were counterstained with DAPI for 10 min. The cells were observed with an EVOS™ M5000 system (Invitrogen), a 10× objective or confocal laser scanning microscope (ZEISS), and a 63× objective.

### 4.9. Co-Immunoprecipitation (Co-IP)

For the co-IP assays, HEK293T cells were transfected with the corresponding plasmids. The cells were harvested by using centrifugation (2000 rpm, 25 °C for 10 min), lysed in NP-40 supplemented with 1 mM phenylmethyl sulfonyl fluoride (PMSF) at 4 °C for 1 h, and then centrifuged at 12,000× *g* for 10 min. Immunoprecipitation was performed using protein A + G agarose according to the manufacturer’s instructions, and then the precleared cell lysates were mixed with ANTI-FLAG^®^ M2 Affinity Gel beads and incubated overnight at 4 °C. The next day, the beads were washed five times with ice-cold PBS and then centrifuged at 1000× *g* for 5 min. The proteins were eluted with elution buffer (5% SDS and 1% TritonX-100) and analyzed by using SDS-PAGE and immunoblotting.

### 4.10. Ubiquitination Assays

The cells were transfected with Ub-HA or its mutant vectors. To prevent proteasomal degradation, cells were treated with 20 μM of MG132 for 6 h before harvest. Thirty-six hours after transfection, cells were harvested, and the following steps were the same as for normal co-IP.

### 4.11. Statistical Analysis

Data were obtained from at least three independent experiments for the quantitative analysis, which were conducted using Student’s *t*-tests for two groups. Statistical analyses were performed using the GraphPad Prism v9 software. Differences considered to be significant at *p* < 0.05 are indicated by * and those considered to be significant at *p* < 0.01 are indicated by **.

## 5. Conclusions

We report a new function for the host E3-ubqiuitin ligase TRIM28 in promoting PRRSV replication. We propose that TRIM28 acts as a stabilizer of PRRSV viral protein GP4 by inhibiting its ubiquitination and degradation to stabilize GP4 protein expression, thereby facilitating PRRSV replication.

## Figures and Tables

**Table 1:** Primer sequences for gene cloning and RT-qPCR.

Primer Name	Sequence (5′–3′)	Purpose
Flag/Myc/HA-TRIM28-For	CAAGCTTATGGCGGCCTCGGCG	Protein expression
Flag/Myc/HA-TRIM28-Rev	GGAATTCTTATCAGGGGCCATCACCAGGG	
TRIM28-RING-For	CCATGGAGGCCCGAATTCGGATGGCGGCCTCGGCGGCG	
TRIM28-RING-Rev	GCCGCGGTACCTCGAGTTATTGCTTGCACACGGGACAG	
TRIM28-BBOX + CC-For	GGAATTCGGATGCAGTGCTTCTCCAAAGACATCG	
TRIM28-BBOX + CC-Rev	CCTCGAGTTAGGTGGCACTGTCATCCAGGGTT	
TRIM28-PHD + BR-For	GGAATTCGGATGATTTGCCGTGTCTGCCAGAAG	
TRIM28-PHD + BR-Rev	CCTCGAGTTATCAGGGGCCATCACCAGGG	
Swine-TRIM28-For	CAGTGCGAGTTCTGCTTCC	RT-qPCR
Swine-TRIM28-Rev	TGCTGTCTCCACCGTCAA	
Swine-β-actin-For	CGGGACATCAAGGAGAAGC	[43]
Swine-β-actin- Rev	CTCGTTGCCGATGGTGATG	
Monkey-TRIM28-For	GAGCCTCTGTGTGAGACCTGTG	
Monkey-TRIM28-Rev	CAAAACAGCACAAGGGGTT	
Monkey-β-actin-For	GGCACCACACCTTCTACAAT	
Monkey-β-actin- Rev	AACATGATCTGGGTCATCTTCTC	
PRRSV-ORF7-For	AAACCAGTCCAGAGGCAAGG	[44]
PRRSV-ORF7-Rev	GCAAACTAAACTCCACAGTGTAA	

**Table 2:** Sequences of siRNAs.

siRNA	Sequence (5′–3′)
NC-For	UUCUCCGAACGUGUCACGUTT
NC-Rev	ACGUGACACGUUCGGAGAATT
siRNA-1-For	CACUAGCUGUGAGGAUAAUTT
siRNA-1-Rev	AUUAUCCUCACAGCUAGUGTT
siRNA-2-For	GGCGAGAUGAAGUUUCAGUTT
siRNA-2-Rev	ACUGAAACUUCAUCUCGCCTT
siRNA-3-For	CUGAGGACUACAACCUUAUTT
siRNA-3-Rev	AUAAGGUUGUAGUCCUCAGTT

## Data Availability

Not applicable.
